# Severe tenosynovitis with rapidly fluctuating power Doppler activity, a clue for the diagnosis of Blau syndrome: a case report

**DOI:** 10.3389/fped.2025.1662832

**Published:** 2025-08-13

**Authors:** Serena Pastore, Carolina Carraro, Eleonora De Martino, Alessia Pin, Irene Bruno, Nunzia Zanotta, Erica Valencic, Alberto Tommasini, Andrea Taddio

**Affiliations:** ^1^ Department of Pediatrics, Institute for Maternal and Child Health IRCCS Burlo Garofolo, Trieste, Italy; ^2^ Department of Medicine, Surgery and Health Sciences, University of Trieste, Trieste, Italy; ^3^ Department of Advanced Translational Microbiology, Institute for Maternal and Child Health IRCCS Burlo Garofolo, Trieste, Italy

**Keywords:** Blau syndrome, ultrasonography, synovitis, NOD2 protein, cytokines, inflammation

## Abstract

**Background:**

Blau Syndrome is a rare monogenic disorder characterized by granulomatous polyarthritis, dermatitis, and uveitis. The diagnosis can be challenging as symptoms may not always align with the classic triad.

**Case description:**

An 8-year-old girl presented with fluctuant swellings in the wrists and ankles, strength reduction and stiffness. Blood tests showed lymphopenia, elevated inflammation markers and positivity of anti-nuclear antibodies. Ultrasound revealed severe tenosynovitis with no power Doppler signal. After fifteen days, she developed fever and widespread joint pain. Laboratory tests detected a marked worsening of inflammatory indices. Musculoskeletal ultrasound showed severe tenosynovitis with a prominent power Doppler signal. A targeted genetic investigation identified a *de novo* pathogenic variant in the *NOD2* gene, confirming the diagnosis of Blau Syndrome. The patient underwent brief treatment with corticosteroids and long-term therapy with methotrexate and adalimumab, achieving good clinical improvement.

**Conclusion:**

The diagnosis was suspected based on severe tenosynovitis of wrists and ankles with power doppler signal fluctuation, despite the absence of other typical Blau Syndrome symptoms. High cytokines levels were observed, which normalized after treatment. Transcriptomic analysis revealed an increased expression of genes related to cellular stress and induction of the TNF pathway.

## 1 Introduction

Blau Syndrome (BS) is a rare autosomal dominant monogenic granulomatous autoinflammatory disorder caused by gain-of-function (GOF) mutations in *NOD2* gene, which encodes nucleotide-binding oligomerization domain-containing protein 2. These mutations cause excessive inflammatory cytokines production. BS is classically defined by the triad of granulomatous polyarthritis, dermatitis, and uveitis. The skin rash typically appears within the first year of life. Between ages 2–4, about 96% of patients develop polyarticular “boggy” synovitis and tenosynovitis affecting peripheral joints of the wrists (87%), knees (73%), ankles (63%), and proximal interphalangeal (PIP) joints of the hands (53%) (1). Uveitis occurs in 80% during early childhood, often involving the posterior chamber of the eye or presenting as panuveitis in 75% (2). However, the diagnosis might be challenging or delayed as non-triad symptoms may be part of the clinical picture. At the same time, the usual signs may not all be concurrently present or may be lacking at all (Table 1) (3).

**Table 1:** Diagnostic criteria of Blau syndrome.

Joint involvement	Arthritis	Small and big joints
	Tenosynovitis	Severe tenosynovitis of multiple tendons
Ocular involvement	Uveitis	Anterior
		Posterior
		Panuveitis
		Episcleritis
Cutaneous involvement	Specific	Lichenoid or granulomatous papules
		Nodules
	Non specific	Ichthyosiform exfoliation
		Pernio, erythromelalgia, acrocyanosis
		Recurrent oral and genital ulcers
		Dermatitis-like eruption
		Chronic urticaria
Constitutional symptoms	Recurrent fever	
Others	Gastrointestinal symptoms	Recurrent abdominal pain
		Nausea and vomiting
	Neurological symptoms	Recurrent headaches, chronic migraine
		Epilepsy
		Abnormal movements

## 2 Case description

An 8-year-old girl was referred by a physiatrist for muscle weakness, nocturnal leg pain and stiffness. Her medical history was remarkable for the presence of fluctuating, non-painful swelling on the dorsal wrists and ankles by the age of 2 years old.

**Figure 1:**
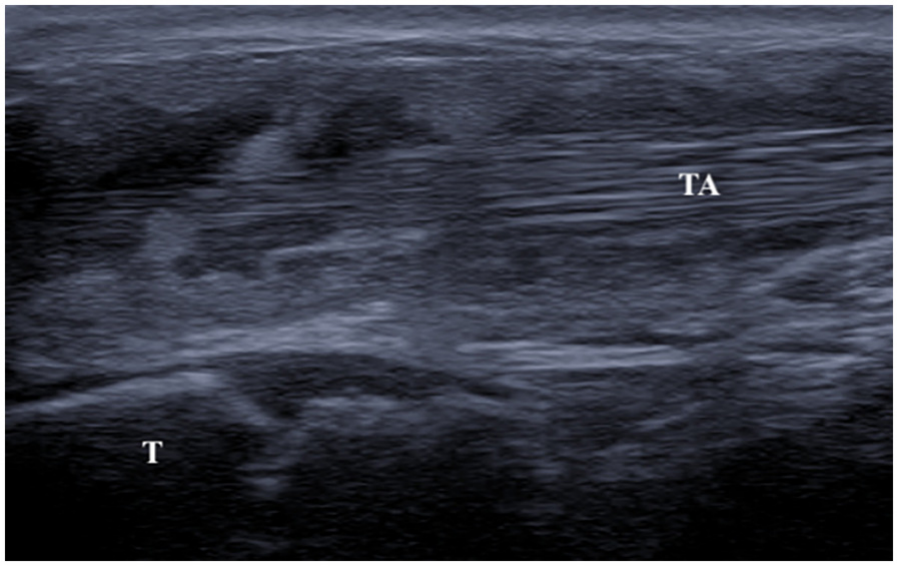
Ultrasound image (longitudinal scan) of anterior tibial tendon. Presence of anechoic peritendinous effusion and severe synovial hypertrophy with a villous pattern. (TA, tibialis anterior; T, tibia).

Ultrasound (US) performed at another center at ages 2 and 4 identified a thickening of subcutaneous tissues, attributed to an abnormal fat distribution. Upon objective examination, she exhibited excellent overall health, was afebrile, had normal cardio-thoracic auscultation, and showed no signs of hepatosplenomegaly or skin lesions. Swelling of wrists and ankles was noted with soft consistency, not warm, and with no joint limitations. She displayed an abnormal gait by dragging of the lower limbs and had difficulty ascending stairs. Laboratory tests revealed lymphopenia (750/mmc), elevated inflammatory markers (ESR 50 mm/h, CRP 10.4 mg/L), and high siglec-1 expression on peripheral blood monocytes, indicating interferon (IFN)-driven inflammation. Antinuclear antibodies (ANA) were positive at 1:1,280 titer, while extractable nuclear antigens (ENA) and rheumatoid factor were negative. The US showed abnormal tenosynovial effusion and synovial hypertrophy with a villous pattern around the tendons in the ankles, wrists, and flexor digitis, graded as 3 by OMERACT criteria (4, 5); but no power Doppler signal was detected (Figure 1). The hands x-ray proved a slight reduction in the representation of the bones in the carpal row. No bone erosions were detected.

Fifteen days after the first examination, the child presented to the emergency department for fever associated with neck stiffness and widespread joint pain, mainly in the wrists and ankles.

**Figure 2:**
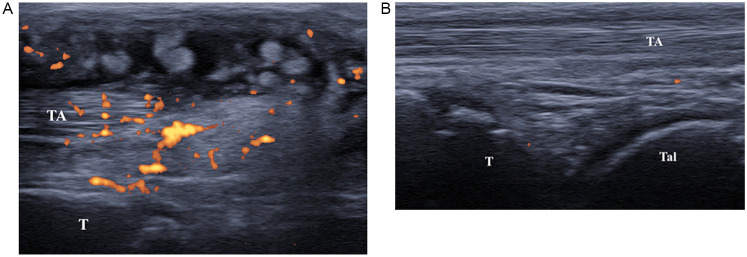
**(A)** Ultrasound image (longitudinal scan) of anterior tibial tendon. Presence of anechoic peritendinous effusion and severe synovial hypertrophy with hyperechoic synovial villi and diffuse power Doppler signal. (TA, tibialis anterior; T, tibia). **(B)** Ultrasound image (longitudinal scan) of anterior tibial tendon. Presence of minimal anechoic peritendinous effusion and minimal synovial hypertrophy. (TA, tibialis anterior; T, tibia; Tal, talus).

Upon evaluation, she was in poor general condition, febrile, with boggy swellings over the extensor aspect of the ankle, wrist joints, and all fingers, with intense pain on mobilization and functional limitation. Marked neck and hip stiffness was also noticed. Musculoskeletal US confirmed severe villous tenosynovitis [Grade 3 according to the OMERACT scoring system (4, 5)] with a strong power Doppler signal (Figure 2A). Hypervascularization was also identified in the non-synovial soft tissues surrounding the tendons, producing a characteristic sonographic appearance known as the “rope on fire” sign (6). On admission, laboratory tests revealed a marked worsening of inflammatory markers: ESR 69 mm/h, CRP 167.7 mg/L, IgG 1,505 mg/dl. Creatine Kinase (58 U/L) and aldolase (6.2 U/L) levels excluded inflammatory myopathy. Neuron-specific enolase (NSE) and fecal calprotectin were negative, ruling out a paraneoplastic condition or inflammatory bowel disease-associated arthritis. Ophthalmological examination, echocardiography and urinalysis were normal. Therefore, our diagnostic pathway started with the detection of severe tenosynovitis in multiple locations. The absence of arthritis made the onset of juvenile idiopathic arthritis unlikely. Consequently, we conducted blood tests, which revealed lymphopenia and elevated inflammatory markers, suggesting an underlying inflammatory condition.

We subsequently assessed the interferon pathway, which showed a markedly increased interferon signature, further supporting our suspicion. The lack of ocular inflammation and cutaneous manifestations made Blau syndrome seem less likely, despite the presence of severe tenosynovitis, a hallmark of the disease.

Given these findings, we performed targeted genetic testing after informed consent, identifying a *de novo* pathogenic variant in the *NOD2* gene (c.1807C > A NM_022162; p.H603N NP_071445), confirming the diagnosis.

Intravenous methylprednisolone 1 mg/kg was started, with a prompt clinical improvement.

After a week of hospitalization, the patient was discharged in good general condition. Corticosteroid administration was switched from intravenous to oral, and subcutaneous biweekly adalimumab (20 mg) and weekly methotrexate (10 mg) were started.

One-month after discharge, the patient showed noticeable clinical improvement. In particular, US showed no effusion around the tendons, which appeared to be surrounded only by minimal synovial hypertrophy with no power Doppler signal (Figure 2B). Also, inflammatory markers (ESR, CRP, and siglec-1) tested all negative, highlighting the dramatic improvement of the disease.

Additionally, post-treatment whole blood RNA and pre- and post-therapy serum samples underwent transcriptomic and cytokine profiling. Differential gene expression analysis was conducted in comparison with healthy controls. Pathway enrichment analysis of differentially expressed genes revealed increased expression of genes related to cellular stress and induction of the TNF pathway.

Cytokine measure assessed using a magnetic bead-based multiplex immunoassay (Bio-Plex®, BIO-RAD Laboratories, Milan, Italy) showed a dramatic decrease in IL1Ra, IL6, and IP10 cytokines following treatment. The concentrations of IL1Ra and IL6 decreased from 1,978.3 and 66.33 pg/ml, respectively, prior to treatment, to values below the detection limit after treatment. The concentration of IP-10 decreased from 9,145.45 pg/ml before to 235.66 pg/ml after treatment.

## 3 Discussion and conclusion

Blau Syndrome is an uncommon monogenic disorder phenotypically defined by the triad of granulomatous polyarthritis, dermatitis, and uveitis.

Gain-of-function mutations in *NOD2* are responsible for this dominantly inherited granulomatous autoinflammatory condition. Pathogenic mutations result in the overexpression of various proinflammatory cytokines, including TNF-alpha, IL-1, and IL-6. In our case, cytokine analysis demonstrated elevated IL-1Ra (indicated increased IL-1 production), IL-6, and IP-10 (reflecting IFN activity), but not TNF-alpha; the levels of all these pro-inflammatory molecules normalized after adalimumab administration. Transcriptomic data obtained on the sample after therapy revealed a hyperactive TNF pathway and upregulation of stress-related genes.

Concerning the clinical features of BS, the skin rash typically manifests within the first year of life, but it may resolve before rheumatologic evaluation. Polyarticular “boggy” synovitis and tenosynovitis are found in 96% of patients aged 2–4 years (1). A distinct differentiation exists between intra-articular synovitis and tenosynovitis, with varying involvement of these two pathological features throughout various subcategories of arthritis. In fact, in BS there is a predominance of the latter, and prominent tenosynovitis in the US can be a valuable clue for diagnosis, like in our case (7). Finally, uveitis develops in 80% of patients early in childhood (2). However, diagnosis may be challenging or delayed due to incomplete triad features or absent classical signs. In our case, the diagnosis was suspected only based on the peculiar ultrasound aspects of the tenosynovitis in the absence of arthritis. This clinical indication was deemed highly indicative of BS, driving prompt confirmatory genetic study by direct sequencing of exon 4 of *NOD2*. A peculiarity of our case is the absence of arthritis, skin rash, and uveitis. Furthermore, while febrile episodes are known to trigger synovitis flares in BS, we emphasize the correlation between this condition with pronounced power Doppler signals in synovial tissue (8). The disease presented a relapse during febrile illness and ultrasonography showed again severe tenosynovitis in the wrists, hands, and ankles, with positive power Doppler signal (grade 3), in contrast to the prior examination, which indicated an absence signal (grade 0). Literature describes minimal synovial power Doppler signal in the youngest and oldest patients and relatively mild signals in treatment patients (9). On contrast, the tenosynovitis with rapidly fluctuating power Doppler activity is unreported in literature and may serve as distinctive diagnostic indicator of BS, highlighting the unique autoinflammatory characteristics of the condition. Musculoskeletal ultrasound combined with clinical assessment by a qualified rheumatologist, is crucial for accurate interpretation emphasizing the need for ultrasound training in pediatric rheumatology.

## Data Availability

The raw data supporting the conclusions of this article will be made available by the authors, without undue reservation.
